# Sequences and animal intelligence

**DOI:** 10.1098/rstb.2024.0116

**Published:** 2025-06-26

**Authors:** Johan Lind, Anna Jon-And

**Affiliations:** ^1^ Biology Division, Department of Physics, Chemistry, and Biology (IFM), Linköping University, Linköping, Sweden; ^2^ Centre for Cultural Evolution, Department of Psychology, Stockholm University, Stockholm, Sweden; ^3^ Department of Romance Studies and Classics, Stockholm University, Stockholm, Sweden

**Keywords:** memory for stimulus sequence, behaviour sequence, sequential behaviour, animal cognition, associative learning

## Abstract

Here, we explore some cognitive mechanisms that support and constrain sequential abilities in non-human animals (hereafter animals). By examining limits in memory for stimulus sequences and how behaviour sequences can be learned, we highlight the combinatorial costs that arise as sequences get increasingly longer, which may hinder the development of cognitive abilities that require faithful representation of sequences, like language. We discuss a trace memory model as a framework for understanding how animals represent stimulus sequences and suggest that animals represent sequences as unstructured collections of decaying memory traces rather than representing order faithfully. The implications of this model challenge traditional interpretations of declarative and rule-based learning in animals. In addition, we explore associative learning models that can account for how animals acquire behaviour sequences without precise memory of stimulus sequences. Current models have proven powerful in accounting for complex behaviour sequences. We end by asking what the value is of anthropocentric models in the study of animal intelligence, if other models provide more accurate predictions of animal behaviour.

This article is part of the Theo Murphy meeting issue ‘Selection shapes diverse animal minds’.

## 1. Introduction

Animals exist within a constant flow of sequences; they perceive sequences of stimuli and they perform sequences of behaviours in response to stimuli. Here we look at limits of memory for stimulus sequences in non-human animals (hereafter animals), and how animals can learn behaviour sequences, and explore how sequential aspects of animal life can help us better understand mechanisms and evolution of behaviour. First, we will present current research that have analysed fundamental costs associated with both remembering sequential information and learning increasingly longer behaviour sequences. Second, we will present new findings on how animals are limited in terms of remembering stimulus sequences. Third, we will ask if remembering stimulus sequences is required for learning behaviour sequences. We will end by discussing theoretical discrepancies in the current literature concerning animal intelligence in the broad sense, touching upon topics such as animal language, episodic memory, planning and social learning. Acknowledging that resource exploitation depends on both the information reaching the animal from the environment and how the animal uses behaviour sequences to manipulate the environment, lies at the core of an ecological approach to cognition, in contrast to simply considering an animal in isolation. But, before we start, let us clarify what kind of sequences we are interested in.

**Figure 1.**
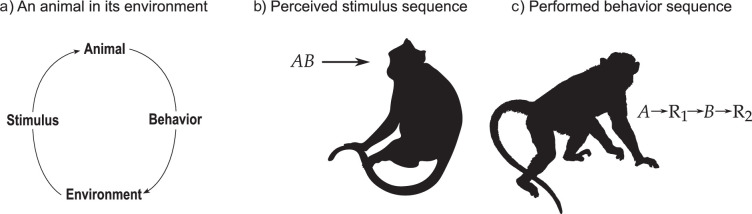
Illustrations of the different sequential aspects of behaviour and cognition explored in this article. (a) We consider an animal and its environment as two interacting dynamical systems, where state variables in the animal determine probabilities for its behaviours, that is the animal’s output. Each behaviour, in turn, puts the environment in a state determining an output stimulus. (b) We are interested in sequences of stimuli, that is temporal sequences of more than one stimulus. We will not discuss single events, or simultaneous presentations of more than one stimulus, such as compound stimuli. (c) When discussing output sequences, we consider sequences in the form where the animal is subjected to a stimulus, for example A, and performs a behaviour, for example $R_{1}$, in response, and so on, to at least two distinctly identifiable behaviours being performed.

We distinguish between input and output sequences (figure 1). We refer to input sequences as temporal sequences of stimuli that reach the animal without an opportunity to respond after each individual stimulus. An example is a stimulus sequence AB that consists of stimulus A, followed by stimulus B one time step later. In the laboratory, this can mean that a pigeon first perceives a green light, and after the green light is turned off it is followed by a presentation of a red light. In a sequence discrimination experiment, the pigeon can collect a reward if it responds by pecking a lever to one of several different stimulus sequences, for example the sequence GreenRed. In one such study, pigeons were rewarded for pecking the lever after having perceived GreenRed, but they were not rewarded after having perceived three other stimulus sequences, namely GreenGreen, RedGreen or RedRed, respectively [1].

Let us now turn to output sequences, that is sequences of behaviour. With a behaviour sequence we mean that an animal performs a behaviour towards a stimulus, which in turn changes the stimulus situation, so that when the animal performs the next behaviour the environment is different than when the animal performed the first behaviour. This can be described as an animal perceiving stimulus A and responding with behaviour R, and we can describe a behaviour sequence as $A\to R_{1}\to B\to R_{2}$. Behaviour sequences can be longer than this. Take the sequence when a chimpanzee (*Pan troglodytes*) opens a nut by striking it with a stone against an anvil. This sequence involves behaviours and physical objects, and it can end with the consumption of a rewarding nut kernel [2]. This sequence can be described as a series of stimuli followed by individual behaviours, such as


$$\begin{array}{c} Nut\to Take\;nut\;\to Nut\;in\;hand\;\to Place\;\;nut\to Nut\;on\;anvil\to Take\;\;stone\to \\ Stone\;in\;hand\;\to Strike\;\to Open\;nut\;\to Eat\;\;nut\;\to Reward \end{array}$$

## 2. Sequences and their costs in the evolution of intelligence

One may easily assume that paying attention to longer stimulus sequences and learning longer behaviour sequences is overall useful. However, focusing solely on benefits of flexibility while ignoring costs may not help us understand variation between species, and the importance of combinatorial costs has been highlighted before [3–5]. The importance of combinatorial costs for the evolution of intelligence is the focus of a recent book on the evolutionary transition from animal intelligence to human cultural evolution [6] and of a theoretical study exploring initial evolutionary steps towards human language and thinking [7].

### (a) Combinatorial costs with sequences of stimuli

Let us start by exploring costs incurred by paying attention to stimulus sequences. A resting hypothetical flycatcher, perched above a park bench, might overhear a conversation and react differently depending on whether a person says ‘flesh flies gather’ or ‘flesh gather flies’. In the first case, the flycatcher might prepare to catch some juicy flies, interpreting it as a call to action. In the second case, it would likely remain at rest, as such a general statement would not motivate immediate action from the flycatcher.

Organisms constantly encounter stimuli and must balance the benefits of exploring and remembering more information for learning and decision-making against exploratory costs and costs of increased memory. Take an animal that lives in a world consisting of n stimuli, that can perceive sequences of length d. For instance, if an animal remembers only the current stimulus (d=1), it must know what to do in n situations. If it remembers sequences consisting of two successive stimuli (d=2), the possible situations to learn about increase to n². As sequence length d increases, the number of scenarios grows exponentially [6]. As Enquist *et al.* [6] pointed out, all potential stimulus sequences are of course unlikely to occur, but if an animal pays attention to longer stimulus sequences, it will face an exponential increase in the number of situations that require a decision. Even with very few stimuli, this becomes a daunting task that can only be solved by ignoring parts of the incoming information. The costs of representing the order of long stimulus sequences can be a key factor that has prevented animals from evolving language and thinking [7].

Let us return to the flycatcher example. Consider that the flycatcher has not heard the expressions ‘flesh flies gather’ and ‘flesh gather flies’ before but is familiar with the individual words. A minimum requirement for immediately understanding that the expressions mean different things is a capacity to represent sequences of length three. The combinatorial explosion caused by remembering sequences can be part of the explanation for why language takes such a long time to learn [6]. It seems unlikely that flycatchers with short lifespans would have evolved the cognitive capacities required for such sequential abilities.

### (b) Combinatorial costs with learning behaviour sequences

There are also combinatorial costs associated with learning behaviour sequences. Let us return to the above example of chimpanzees learning to crack open nuts using stone tools. Observations of the ontogeny of stone tool use behaviour in the wild showed that it took nearly 4 years for young chimpanzees to learn actual nut cracking by putting the five basic actions together in an adequate behavioural sequence [2]. Initially, only single actions were recorded, and behaviour sequences increased in length throughout the study period. The development of this behavior sequence took a long time, it was gradual, and a lot of exploration was needed.

Combinatorial costs of behavioural output have been explored recently in some detail [6,8,9]. Exploring the world becomes increasingly costlier as behaviour repertoires become larger and behaviour sequences become longer. Here, consider an animal with m behaviours in its repertoire, which can explore sequences of behaviour that are l behaviours long. Through brute-force exploration, the animal can try out $m^{l}$ sequences. Among all these possibilities, if r sequences result in a reward, the anticipated number of trials before a rewarding sequence found is $m^{l}/r$. Without favourable learning conditions or genetically supported learning, longer sequences that are rewarding will be exponentially more difficult to find than shorter sequences. Enquist *et al.* [6] exemplified the learning costs of longer behaviour sequences with the following example. To find a rewarding sequence of three consecutive behaviours for an animal with a repertoire of only 10 behaviours would take $10^{3}=1000$ attempts. If the same animal should find a rewarding behaviour sequence of 10 behaviours, it would be expected to require $10^{10}=10$ billion attempts. There are several ways in which these learning costs can be reduced, for example through genetic guidance of learning [8,10], social contexts that pave the way for favourable learning opportunities [11,12] or through cumulative culture combined with institutionalized learning in current educational systems.

## 3. Limited memory for stimulus sequences

Regardless of field—psychology [1,13,14], neuroscience [15–18], language evolution [19,20] or rule learning [21–24]—the observation that animals only represent sequences of stimuli approximately, and not through faithful symbolic representations, stands out [25,26]. Recent studies suggest a trace memory model as a compelling explanation for such approximate representations and variability in performance [6,25].

According to this model, memory of a recently perceived stimulus decays rapidly, disappearing within seconds to minutes. Applied to a stimulus sequence, the model suggests that animals represent each stimulus with varying intensity based on the time since its presentation. For example, if an animal first sees a green light and then a red light, the memory trace of the red light will be stronger due to its more recent exposure, while the trace of the green light will have decayed more. A meta-analysis of over 100 experiments on memory for stimulus sequences supports this model; animals appear to treat sequences as unstructured collections of decaying memory traces, without representing exact order [25]. Trace memory not only allows approximate sequence discrimination but also helps isolate relevant stimuli from noise. In fact, a trace memory model is more efficient than one that represents sequences faithfully when the task is to learn to respond to stimuli from a few time steps back and ignore more recent stimuli [7]. A trace memory is thus compatible with animals being able to, for example, categorize sequences based on their initial part [27] or identify non-adjacent dependencies [28].

Despite its explanatory potential, trace memory models are seldom referenced in studies on how animals represent sequential input. We argue that incorporating the trace memory model could significantly enhance our understanding of phenomena that depend on how animals represent sequences of stimuli. Consider an example from an artificial grammar study by van Heijningen *et al.* [24]. In this study, zebra finches (*Taeniopygia guttata*) were exposed to sound sequences and trained to respond to ABA and BAB but not to AAB, ABB, BAA or BBA. To test whether the birds had learned a rule distinguishing sequences that start and end with the same stimulus from those that do not, the researchers introduced new sequences: ABBA, BAAB, ABAB and BABA. Contrary to expectations, the zebra finches largely avoided responding to ABBA and BAAB, treating them similar to AAB, ABB, BAA and BBA, and responded more to ABAB and BABA, treating them as similar to ABA and BAB.

This led the researchers to hypothesize that the finches might instead be focusing on the presence or absence of repeated stimuli (e.g. AA or BB). To test if the birds could shift to a new rule, the researchers conducted a new training phase with sequences that could not be distinguished based on element repeats. The birds were now trained to respond to ABBA, BAAB, ABA and BAB (where first and last stimuli are the same), while ignoring ABAB, BABA, AABB, BBAA, AAB, BBA ABB, BAA (where first and last stimuli are different). This new training set led to a significant decline in the birds' performance. Further probes of the only two individuals that met the learning criteria suggested they relied more on rote memorization of single stimuli rather than applying any rule, rendering results inconclusive.

This excellent study [24] was meticulously designed to distinguish between different rules. However, the absence of consideration for the memory trace model may have led to an oversight of the possibility that the birds’ behavior was not based on rule learning at all.

To illustrate this, let’s examine what the memory trace model predicts for the same experiments, as outlined by Ghirlanda *et al*. [25]. First, the memory trace model accurately predicted the performance of the birds when discriminating ABA and BAB (response) from AAB, ABB, BAA and BBA (no response). Second, the subsequent lack of responses by the zebra finches to ABBA and BAAB is precisely what the trace memory model would expect, as the three last stimuli of both sequences (*BBA* and *AAB,* respectively) are identical to sequences they have been trained not to respond to. Similarly, it is expected that the birds respond to ABAB and BABA, as the last three stimuli (*BAB* and *ABA*) correspond to sequences they have been trained to respond to. Third, when the birds were trained to distinguish sequences that left very similar memory traces, such as *ABBA* and *BBA*, they generally failed. Finally, the memory trace model also predicts the birds’ overall difficulty to generalize rules to novel stimulus sequences—an outcome that contradicts rule-learning.

In this example, the memory trace model makes more accurate predictions and requires fewer assumptions than explanations based on rules or grammar learning. Similarly, many other studies on rule learning, artificial grammar and sequence discrimination have been found to be better explained by a trace memory model [25]. This raises doubts about whether terms like ‘grammar’ or ‘rules’ accurately describe what animals learn about stimulus sequences, suggesting that parallels to human grammar learning might be misleading. While some studies suggest rule generalization to unknown stimuli, the evidence remains inconclusive and may be due to stimulus similarity [28]. Notably, findings from budgerigars [29], often cited as examples of such rule generalization [28], are based on a training procedure, where correct responses are rewarded, rather than on a test procedure. Thus, we cannot draw clear conclusions from this part of the study. Data from test procedures were also presented in that study, and these data are included in Ghirlanda *et al*.’s [25] meta-study and are consistent with predictions made by the trace memory model. Therefore, the trace memory cannot be dismissed as the underlying mechanism, and further research should address this possibility.

## 4. Does learning behavior sequences require memory for stimulus sequences?

We would also like to highlight that studies can benefit from considering knowledge about how animals learn behaviour sequences. We will first present a model for learning behaviour sequences. Then we will outline a recently published study on birds that presented interesting tests on the recursion hypothesis [30]. When we speak of behaviour sequences, we refer to a series of events in which an animal perceives an identifiable state, responds with a behaviour that leads to a new state, and from there may again perform another behaviour, thereby continuing the sequence (figure 1a). Typically, animals experience states that may consist of many components, for example a combination of social and non-social stimuli. When an animal experiences such a compound stimulus, it can potentially learn about both the social and the non-social part of the stimulus, as well as the compound itself. Taking compound and configural information into account can result in increased behavioral complexity [12,31].

Animals performing behaviour sequences is ubiquitous, bears seek out and open trash cans [32], gulls take bags of crisps and tear them open [33] and fish can, through a series of actions, drive a vehicle to reach a pink target [34]. It is notable, however, that bears, gulls and fish are not born with tendencies to seek out trash cans, bags of crisps or pink targets, respectively. How can such behaviour sequences be performed, directed to biologically irrelevant stimuli without faithful representations of stimulus sequences?

The key lies in the fact that animals can both learn about the value of behaviour and the value of stimuli, and Skinner introduced the term chaining for the process of learning behaviour sequences [35]. Modern associative learning models can acquire optimal behaviour in complex environments by including a decision-making mechanism and two types of memory for these processes: one memory tracks the association between a behaviour and a stimulus, while the other estimates the value of a stimulus [8,36]. The model learns behaviour sequences by linking individual behaviours through conditioned reinforcement, where neutral stimuli such as bags of crisps and pink targets that precede primary rewards become reinforcers themselves [37,38]. For example, in the gull example above, the gull learns to find the bag rewarding and will subsequently perform behaviours just to see another bag. It is said that the bag of crisp has become a conditioned reinforcer. In terms of learning, social stimuli can be treated just like any other stimulus, enabling animals to learn sequences of behavior in social settings as well [12,39].

Such computational models of animal learning can today explain the development of behaviour sequences without needing precise representations of stimulus sequences. They have been shown to account well for established learning phenomena in experimental psychology [36,40] and to reproduce patterns of how various behaviour sequences can be acquired in non-human animals, for example in tool use [8], planning [41], social learning [12] and establishing episodic-like memories useful for caching behaviour [42]. Storing information about both stimuli and one’s own behaviours long-term makes evolutionary sense, as it broadens the range of learnable behaviors without adding combinatorial costs [43].

Now, let us examine a recent study that tested the recursion hypothesis on birds learning behavioural sequences [30]. This hypothesis suggests that only humans are capable of embedding element structures within other element structures, a distinctive feature of human language [44]. This means that expressions can be extended potentially infinitely, so that in the sentence ‘The killer-wug the wug-killer followed escaped’, the inner clause ‘the wug-killer followed’ is embedded in ‘The killer-wug escaped’.

In this study, the authors did not consider results that may be caused by associative learning of behaviour sequences [45]. We selected this study as an example of not fully addressing modern associative learning models because it is of high standard in every other aspect, it addresses a timely question and was published in a prestigious journal. Liao *et al.* [30] used two crows (*Corvus corone*) to test the recursion hypothesis. First, they were trained to peck at four different kinds of brackets, presented on a touchscreen, in a specific order. The symbols, made up of different kinds of brackets, some of which were surrounded by boxes, were presented simultaneously in random locations, and one sequence consisted of the following four symbols: {()}. (Please disregard the use of brackets, which are often used to indicate recursion, as crows are not schooled in this notation.) After pre-training, the crows were subjected to other sequences of similar symbols, for example [()] or ([]). Analyses showed that behaviour sequences produced by the crows when subjected to test sequences more often followed a centre-embedded pattern, such as [()], than a crossed pattern [(]) or a tail-embedded pattern [](). The authors rejected the recursion hypothesis (according to which only humans are capable of recursion) and concluded that crows possess recursive abilities, and that recursive abilities may have evolved in the tree of life more than once. However, when Rey & Fagot [45] examined this procedure carefully, they noted that the observed behaviour fits with an alternative and simpler explanation; that the procedure allowed the crows to perform centre-embedded behaviour sequences through associative learning.

There were two key features of the procedure that makes associative learning of centre-embedded behaviour sequences likely [45]. Rey & Fagot [45] noted first that the last two, but never the first, symbols were always surrounded by a box, and that crows therefore learned to avoid pecking at these bordered symbols at the beginning of behaviour sequences. As test trials in experiment 1 consisted of four pecks, another question is what can explain the behaviour during peck three and four, respectively. Rey & Fagot [45] made the point that when animals learn behaviour sequences, they are sensitive to associations between adjacent stimuli. Thus, if the second symbol was ( they were trained to peck ), or if the second symbol was [ they were trained to peck ]. The fourth symbol to peck was then given by prior training, as crows had systematically been trained to not peck symbols more than once per sequence. The procedures and results from experiments 2 and 3 are similar to experiment 1 and are likely to share the same explanation as experiment 1. The nature of how animals can learn behaviour sequences through associative processes can thus explain how recursive patterns, in terms of producing centre-embedded behaviour patterns, emerges as a by-product of well-established associative processes [46].

## 5. Discussion

We have highlighted research on sequential aspects of animal intelligence and that costs can grow exponentially when animals start paying attention to stimulus sequences and when they learn increasingly longer behaviour sequences [6,7,43].

The fact that animal memory encodes stimulus sequences poorly raises the question of how we should interpret behavioural observations that are preceded by stimulus sequences. For instance, despite over 20 years of animal artificial grammar studies [47], Petkov & ten Cate noted in their recent review that current evidence from animal experiments does not conclusively demonstrate faithful and well-structured learning of sequences [28]. Limits in animals’ memory for stimulus sequences highlighted in this text suggest that future studies in artificial grammar may continue to produce results that are not entirely conclusive. This is likely to apply to other human phenomena that require faithful representation of sequential information, such as imitation of sequences, episodic memory and causal learning (see table 1 in [25]).

In addition, models of associative learning predict well how animals can efficiently learn behaviour sequences, if learning conditions permit [8]. This in turn raises questions about how to interpret studies where this is neglected. We exemplified above with a study that interpreted crow behaviour as evidence for recursive capacities [30]. However, when analysed in detail it was found that the crows’ recursive behaviour probably emerged through associative sequence learning [45]. This example touches upon other studies where animals have learned behaviour sequences. There are today many studies that have received considerable attention for various claims, that when analysed by others have been suggested to be better explained by associative learning models. These include, for example, studies on planning [41,48–50], tool use [8,51–53] and causal reasoning [54–56].

We have not delved into detail on what evolutionary paths may lead to faithful sequence representation. Focusing solely on a dichotomy between single and sequential stimuli can lead to an oversimplified view of how animals represent and learn about more complicated, simultaneous stimulus configurations—such as how animals represent compound stimuli. It has been suggested that learning about non-sequential stimulus configurations represents a transitional stage between simpler stimulus-response learning and the ability to represent stimulus sequences and flexibly learn behaviour sequences [31,57,58]. In fact, configural learning theory has successfully explained phenomena that simpler elemental theories cannot [59]. However, as the ability to learn complicated and nonlinear discriminations is widespread in the animal kingdom, from insects [60] to birds [61,62] and mammals [63], it may reflect an important stage in the early evolution of learning, well before vertebrates evolved, but not necessarily a recent phenomenon of relevance for the human evolutionary transition [6].

We want to end by asking if theorizing in studies on animal cognition has become too anthropocentric. Let us clarify our point. When we sit behind our desks, our thought processes are declarative and rule-based at large. Using our own thinking as a model for animal minds will be misleading if other animals do not engage in declarative and rule-based processes that require faithful sequence representations. Take the recent study on counting in crows [64] and consider that such verbal counting tracks progress by counting sequentially aloud or mentally. Such counting and knowledge of integers are cultural traits that only develop in humans after years of practice and lots of social input [6]. Is the concept of counting a useful description for when trained crows are asked to vocalize two times and respond by ‘cawing’ two times in two thirds of the trials, and in the remaining trials, the crows ‘cawed’ one or three times. If we do detect systematic differences in how humans and other animals deal with input and output sequences, why continue to use human sequential abilities as the default model for other animals, when other models exist that make better predictions?

## Data Availability

This article has no additional data.
